# Supplementary material for: Development of a Rapid and Sensitive Fluorescence Sensing Method for the Detection of Acetaldehyde in Alcoholic Beverages
Source: Foods. 2022 Oct 31;11(21):3450. doi: 10.3390/foods11213450 (PMC9655822; doi:10.3390/foods11213450)
Supplement: Supplementary file 1 [file foods-11-03450-s001.zip › foods-1945430-supplementary.pdf]

foods

MDPI

Article

Development of a Rapid and Sensitive Fluorescence Sensing Method for the Detection of Acetaldehyde in Alcoholic Beverages

Yisong Liu [1,2], Chunfeng Liu [1,2,*], Xin Xu [1,2], Chengtuo Niu [1,2], Jinjing Wang [1,2], Feiyun Zheng [1,2], Qi Li [1,2]

[1] Key Laboratory of Industrial Biotechnology, Ministry of Education, School of Biotechnology, Jiangnan University, Wuxi 214122, China
[2] Laboratory of Brewing Science and Technology, School of Biotechnology, Jiangnan University, Wuxi 214122, China
* Correspondence: Liuchunfeng@jiangnan.edu.cn (C.L.); Tel.: +86--0510-85918176

Supplementary Materials

Table S1. The basic information of real samples of beer

The sample number of beer	Type	original gravity (°P)	Alcohol by volume (v/v%)
1	commercial lager	10.5	4.2
2	commercial lager	11.0	4.3
3	commercial lager	13.0	5
4	fermentation broth	12.0	4.6
5	fermentation broth	12.0	4.9
6	fermentation broth	12.0	5.2

Table S2. The basic information of real samples of liquor

The sample number of liquor	Flavor	Alcohol by volume (v/v%)
1	Jiang-flavor	45.0
2	Strong-flavor	52.0
3	Mild-flavor	42.0
4	Rice-flavor	38.0
5	Zhima- flavor	39.0
6	Laobaigan flavor	56.0

Table S3. The basic information of real samples of *Huangjiu*

The sample number of *Huangjiu*	Type of sweetness	Residual sugar content (g/L)	Alcohol by volume (v/v%)
1	Sweet	135.0	11.0
2	Sweet	106.0	10.0
3	Semi-dry	31.0	14.0
4	Semi-dry	26.0	17.0
5	Dry	11.0	13.0
6	Dry	13.0	14.0

Table S4. The basic information of real samples of wine (red wine)

The sample number of wine	Type of sweetness	Residual sugar content (g/L)	Alcohol by volume (v/v%)
1	Sweet	58.0	12.0
2	Semi-sweet	35.0	13.0
3	Semi-dry	8.0	13.0
4	Semi-dry	9.0	14.0
5	Dry	3.0	13.0
6	Dry	2.0	15.0

Table S5. Concentration of analytes of the anti-interference analysis in different kinds of model alcoholic beverages.

Analytes	Concentration in model beer (mg/L)	Concentration in model liquor (mg/L)	Concentration in model Huangjiu (mg/L)	Concentration in model wine (mg/L)
acetaldehyde	10	300	50	100
acetal	25	800	50	100
propionaldehyde	0.3	20	5	10
butyraldehyde	0.3	10	5	10
isobutyraldehyde	0.3	100	5	10
isovalerealdehyde	0.1	-	-	-
heptanaldehyde	0.2	-	-	-
benzaldehyde	-	400	150	-
phenylacetalde-hyde	-	440	-	-
octanal	0.2		-	-
furfural	2	20	10	20
5-HMF	8	600	500	-
2.3-butanedione	0.1	230	-	2
2.3-pentanedione	0.5	-	-	-
acetoin	5	-	-	5
methyglyoxal	0.1	-	-	-
1-propanol	25	2250	80	-
isoamyl aclcohol	100	460	300	150
isobutanol	-	-	100	150
3-methylbutanol	-	-	200	-
β-phenylethanol	-	-	100	-
lactic acid	-	1000	5000	500
acetic acid	20	1000	1500	2000
caproic acid	-	500	-	-
ethyl acetate	50	5000	100	100
isoamyl acetate	10	-	-	-
ethyl caproate	-	3000	500	-
ethyl lactate	-	2000	500	100
pyruvate	100	-	50	20
TMP	-	5	-	-
MES	-	-	180	-
Asn	15	2	-	20
Gln	10	-	-	1.5

* ″-″ means the concentration of the analyte was not reported in the related literature.

Table S6. Principle, applicable samples, linearity range, LOD, recovery and time of detection of the fluorescence sensing method proposed in this study and the previous sensing methods for detecting acetaldehyde in alcoholic beverages.

Method	Principle	Applicable samples	Linearity range (mg/L)	LOD (mg/L)	Recovery (%)	Time of detection (min)
Fluorescence sensing method in this work	PET	Beer, liquor, Huangjiu and wine	0.0053-200	0.0016	92.1-108.1	20
Fluorescence sensing method 1 [23]	-	Wine and vodka	3.1-100	0.9	91-98	30
Fluorescence sensing method 2 [17]	AIE	Liquor and spirits	0-0.044	0.002	96.4-105.7	20

Table S7. Pretreatment, time of pretreatment, linearity range, LOD, recovery and time of detection of the fluorescence sensing method proposed in this study and the previous GC methods for detecting acetaldehyde in alcoholic beverages.

Method	Pretreatment	Time of pretreatment (min)	Linearity range (mg/L)	LOD (mg/L)	Recovery (%)	Time of detection (min)
Fluorescence sensing method in this work	BRM	2	0.0053-200	0.0016	94.0-108.1	20
GC-FID [28]	-	-	0.03-32.2	0.01	79.8-118.2	47
HS-SPME-GC-FID [37]	HS-SPME	15	1-10	0.039	95.1	28
Static HS-GC [38]	HS	35	0.5-2.1	0.5	-	25
HS-ITEX-GC-MS [7]	HS	25	3.9-49.4	0.03	85	29
HS-SPME-GC-MS [39]	HS-SPME	16.8	-	-	-	-
HS-GC-FID [40]	HS	15	5-500	5	93.3-103.8	32

* ”-” means the information was not reported in the related literature.

Figure S1. Distillation apparatus and determination of the optimal concentration ratio. (a) Distillation apparatus 1. (b) Distillation apparatus 2. (c) The relationship between the concentration multiple and the recovery of acetaldehyde.
Figure S2. Anti-interference analysis before distillation (without BRM).
Figure S3. Anti-interference analysis after distillation (with BRM).
Figure S4. Job's plot obtained from fluorescence intensity of FPN1 and acetaldehyde
Figure S5. Sensing mechanism of FPN1 to acetaldehyde. (a) Optimized structures and frontier molecular orbitals (HOMO and LUMO) of FPN1 and FPN1-CH3CHO. (b) The HOMO and LUMO of FPN1-CH3CHO at ground state and excited state.
Figure S6. The calibration curve of fluorescence intensity with the concentration of acetaldehyde.
Figure S7. Consistency test of the fluorescence sensing method with BRM and GC method based on Blant-Altaman..

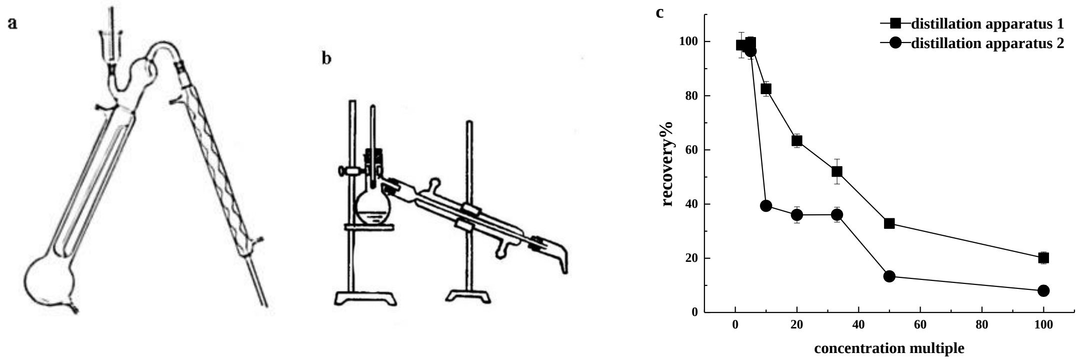

Figure S1. Distillation apparatus and determination of the optimal concentration ratio. (a) Distillation apparatus 1. (b) Distillation apparatus 2. (c) The relationship between the concentration multiple and the recovery of acetaldehyde. All the tests were performed in triplicate.

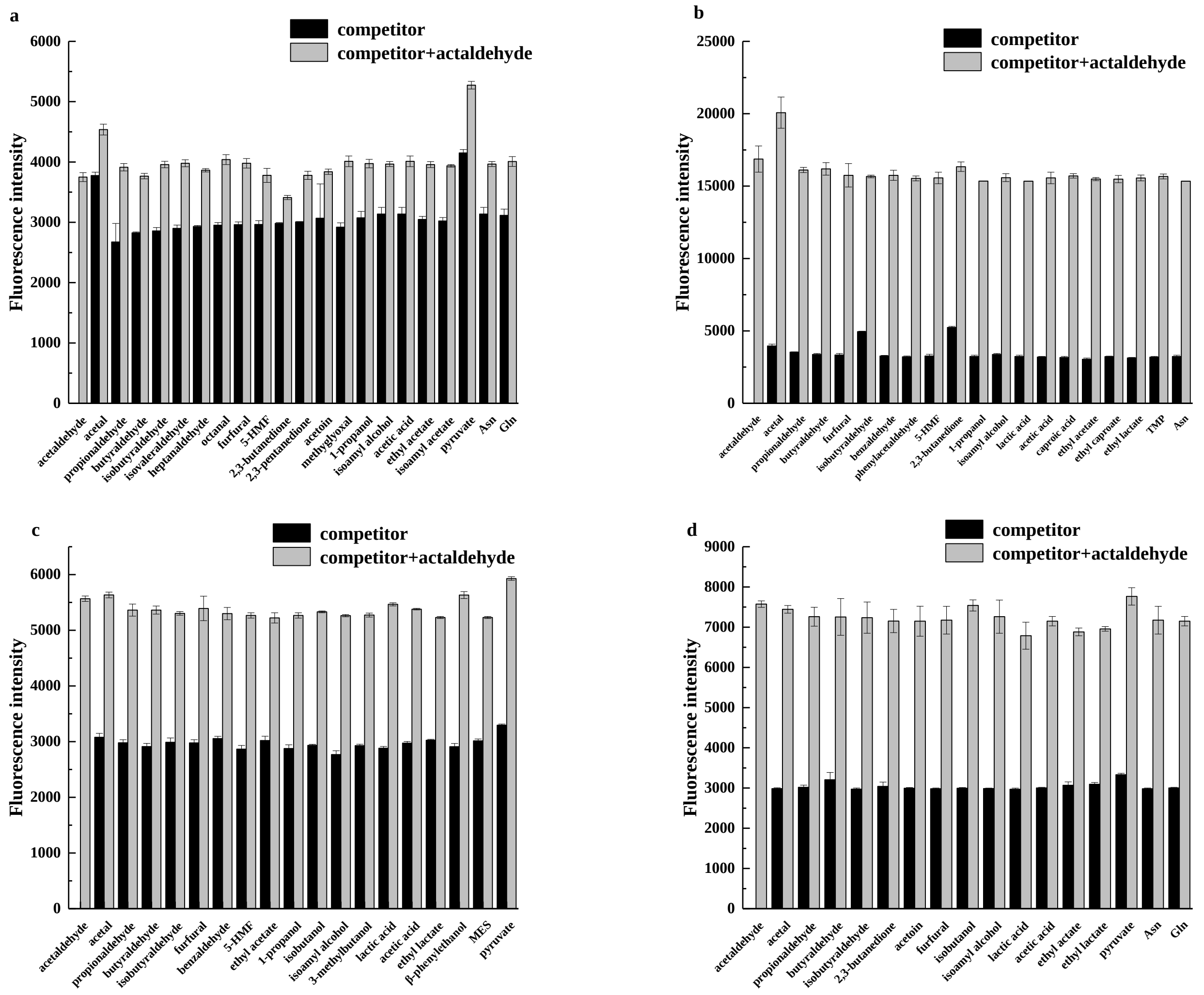

Figure S2. (a) Anti-interference analysis of beer without BRM (b) Anti-interference analysis of liquor without BRM. (c) Anti-interference analysis of *Huangjiu* without BRM. (d) Anti-interference analysis of wine without BRM. The concentration of acetaldehyde and other analytes are showed in Table S6. All the tests were performed in triplicate.

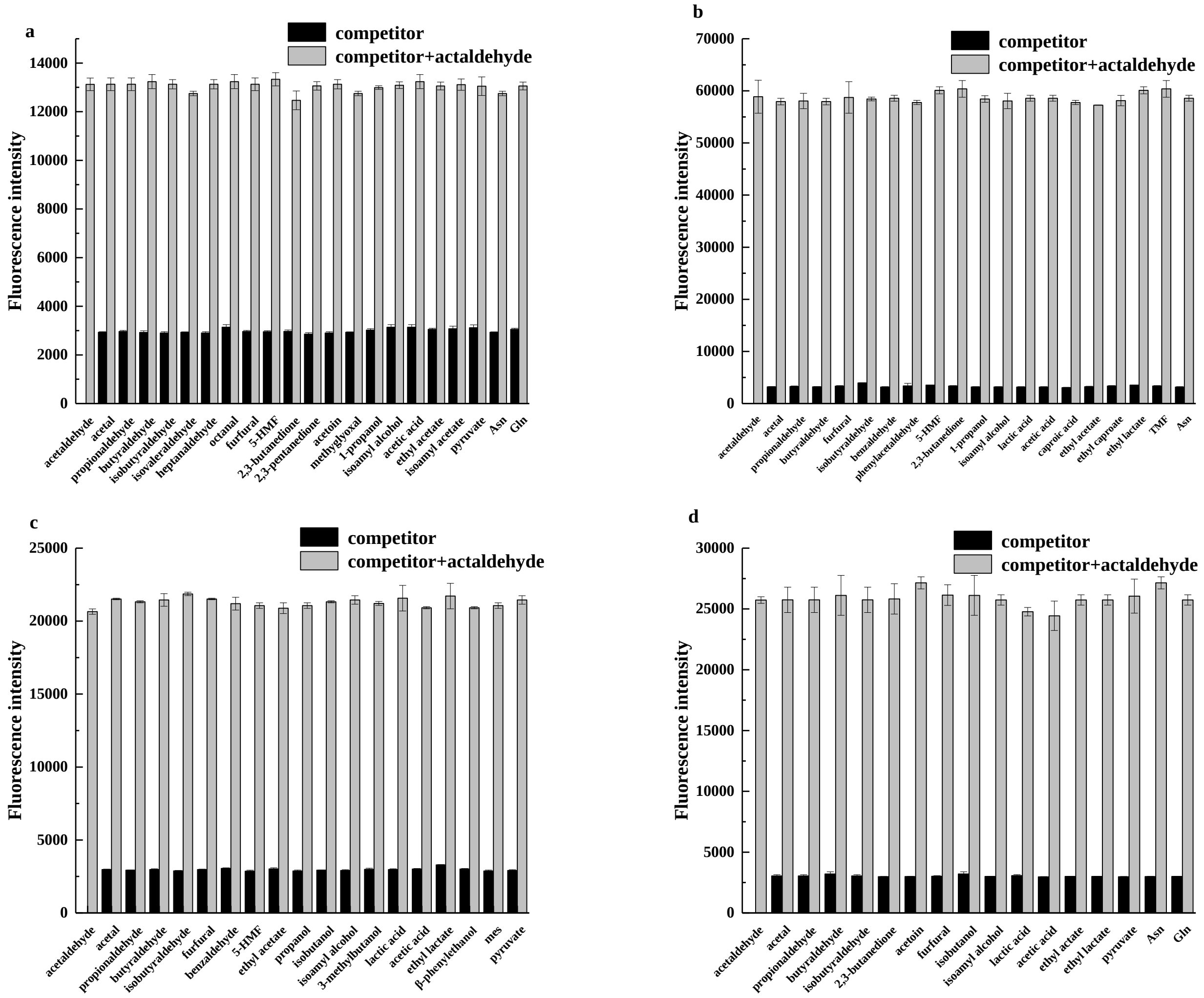

Figure S3. (a) Anti-interference analysis of beer with BRM. (b) Anti-interference analysis of liquor with BRM. (c) Anti-interference analysis of Huangjiu with BRM. (d) Anti-interference analysis of wine with BRM. The concentration of acetaldehyde and other analytes are showed in Table S6. All the tests were performed in triplicate.

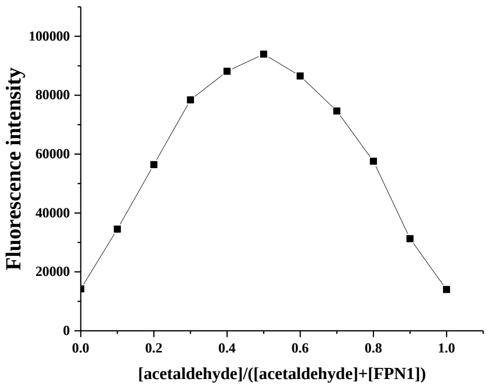

Figure S4. Job's plot obtained from fluorescence intensity of FPN1 and acetaldehyde. All the tests were performed in triplicate.

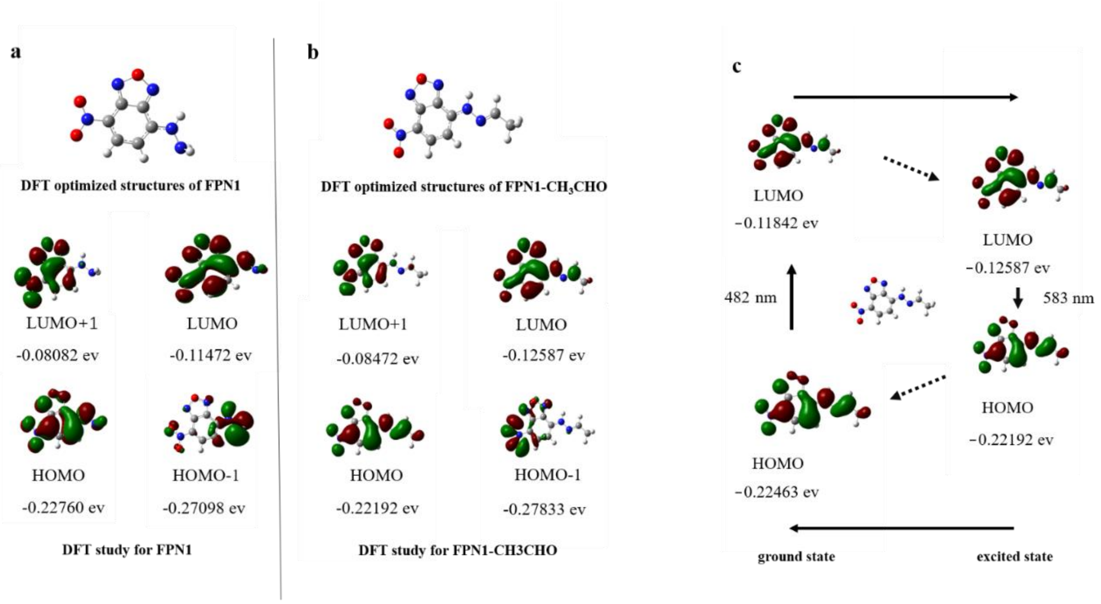

Figure S5. (a) Optimized structures and frontier molecular orbitals (HOMO and LUMO) of FPN1. (b) Optimized structures and frontier molecular orbitals (HOMO and LUMO) of FPN1-CH_3CHO. (c) The HOMO and LUMO of FPN1-CH_3CHO at ground state and excited state.

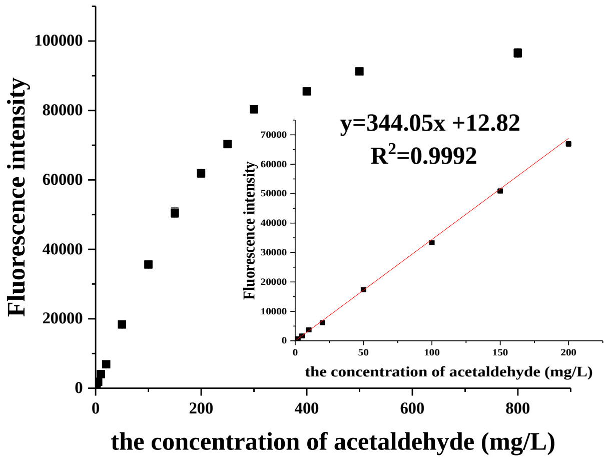

Figure S6. The calibration curve of fluorescence intensity with concentration of acetaldehyde. All data was recorded 20 min after the addition of acetaldehyde. All the tests were performed in triplicate.

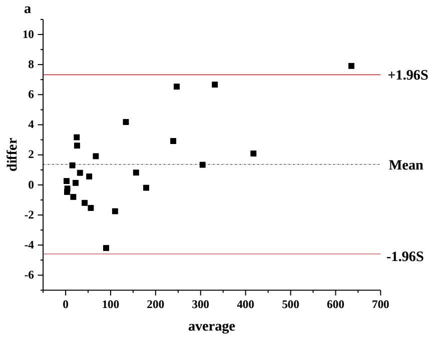

Figure S7. Consistency test of the fluorescence sensing method with BRM and GC method based on Blant-Altaman.
